# Frugivorous Bats Maintain Functional Habitat Connectivity in Agricultural Landscapes but Rely Strongly on Natural Forest Fragments

**DOI:** 10.1371/journal.pone.0120535

**Published:** 2015-04-01

**Authors:** Simon P. Ripperger, Elisabeth K. V. Kalko, Bernal Rodríguez-Herrera, Frieder Mayer, Marco Tschapka

**Affiliations:** 1 Museum für Naturkunde, Leibniz-Institut für Evolutions- und Biodiversitätsforschung, Berlin, Germany; 2 Institute of Experimental Ecology, University of Ulm, Ulm, Germany; 3 Smithsonian Tropical Research Institute, Balboa, Panama; 4 Escuela de Biología, Universidad de Costa Rica, San Pedro, Costa Rica; 5 Berlin-Brandenburg Institute of Advanced Biodiversity Research, Berlin, Germany; Università degli Studi di Napoli Federico II, ITALY

## Abstract

Anthropogenic changes in land use threaten biodiversity and ecosystem functioning by the conversion of natural habitat into agricultural mosaic landscapes, often with drastic consequences for the associated fauna. The first step in the development of efficient conservation plans is to understand movement of animals through complex habitat mosaics. Therefore, we studied ranging behavior and habitat use in *Dermanura watsoni* (Phyllostomidae), a frugivorous bat species that is a valuable seed disperser in degraded ecosystems. Radio-tracking of sixteen bats showed that the animals strongly rely on natural forest. Day roosts were exclusively located within mature forest fragments. Selection ratios showed that the bats foraged selectively within the available habitat and positively selected natural forest. However, larger daily ranges were associated with higher use of degraded habitats. Home range geometry and composition of focal foraging areas indicated that wider ranging bats performed directional foraging bouts from natural to degraded forest sites traversing the matrix over distances of up to three hundred meters. This behavior demonstrates the potential of frugivorous bats to functionally connect fragmented areas by providing ecosystem services between natural and degraded sites, and highlights the need for conservation of natural habitat patches within agricultural landscapes that meet the roosting requirements of bats.

## Introduction

Land-use changes are identified as the major threat to biodiversity, with the most devastating impacts observed in tropical biomes [1]. Conversion of natural habitats to arable or commercially usable land leads to habitat fragmentation and the creation of mosaic landscapes. Consequences for flora and fauna include community changes in species diversity or abundance [2, 3] and breakdowns of relationships among plants and their dispersers, which are followed by changes in seed bank composition [4–6]. Finally, populations experience loss of genetic diversity and genetic differentiation [7] that may derive from altered animal movement patterns [8].

Understanding animal movement is crucial for developing effective landscape level conservation strategies, as movement is a key to successful foraging, mating, or dispersal [9]. However, heterogeneous landscapes are composed of more or less hospitable habitat patches within a non-uniform matrix area. Many studies have shown that the matrix is more accessible to a species the more similar it is structurally to its natural habitat [10]. The response of animals to habitat boundaries may vary among species that evolved either in patchy or in continuous environments and may lead to different levels of susceptibility to habitat fragmentation [8]. Such findings are important to model probability of individuals of a species moving through complex landscapes; yet, such simulations strongly depend upon real data on animal movement and ecology to lay a biological foundation [8, 11].

Telemetry studies contribute to the understanding of human impact on animals and provide essential base information for conservation initiatives [12]. Phyllostomid bats are especially valuable to endangered ecosystems by providing ecosystem services, namely pollination and seed dispersal [13]. Frugivorous phyllostomids and birds play an important role in dispersing seeds to disturbed areas because often other more sensitive fruit-eating taxa have been reduced in abundance by fragmentation itself or by activities such as hunting [14, 15]. Information on habitat use of frugivorous bats is desirable to understand the consequences of human impact, as has been accomplished for other taxa [12]. Most radio-tracking studies on phyllostomid bats in the context of habitat fragmentation either focus on naturally fragmented habitats [16–18] or on anthropogenically modified systems that are composed of forest islands within a strongly contrasting water matrix [19, 20]. The latter work on smaller bats confronted with a water matrix suggests that such species are limited in their foraging range and can only cross narrow habitat disruptions. However, mark-recapture studies in agricultural landscapes provided evidence that several frugivorous and nectarivorous phyllostomid bat species are able to move through mosaic areas while heavily using riparian forests, live fences consisting of planted posts, or corridors [21, 22]. A radio-tracking study in an anthropogenically dominated area showed that two phyllostomid bat species are able to incorporate restored forests into their foraging range [23]. Shade-cocoa plantations may even harbor a more abundant and diverse bat fauna than native forest tracts, if the agroforest sites are neighboring with forest [24]. The evaluation of movement patterns in the landscape context helps to gain a deeper understanding of how bats use distinct elements within highly heterogeneous environments.

In the present study we conducted radio-tracking on frugivorous bats inhabiting an agriculturally dominated area in Costa Rica. We focused on the small phyllostomid bat species *Dermanura watsoni* since it is a valuable organism for fragmented areas. It remains abundant in forest fragments and feeds on fruits of a wide variety of plant species [14, 25]. Its abundance and associations with a large number of plant species make it an important agent for seed dispersal, especially in areas under human influence where large-bodied seed dispersers (e.g., guans, agoutis, or peccaries) experience high hunting pressure [6, 14]. However, populations of *D*. *watsoni* are prone to loss of genetic diversity in such an agricultural landscape when faced with low habitat connectivity [26]. Since gene flow requires successful movement across a landscape *D*. *watsoni* may be limited in its movement capacity by habitat fragmentation [26]. Therefore, we investigate whether *D*. *watsoni* preferably uses natural forest remnants for foraging and roosting and to what extent anthropogenically modified areas will be visited. Furthermore, we examine the reasons for intra- and inter-individual variability in daily range sizes. Finally we draw conclusions from movement patterns for functional landscape connectivity.

## Methods

### Study area

Field work was carried out from January to June 2011 near Reserva Biologica Tirimbina (RBT) in the northern Caribbean lowlands of Costa Rica (10°25’ N, 84°05’ W) at elevations ranging from 100–300 m. The landscape experienced high rates of forest clearing up until the 1980s that caused strong fragmentation of the landscape. Since the 1990s, after the establishment of an innovative conservation program (payments for environmental services), a significant drop in deforestation rates has been observed [27].

**Fig 1:**
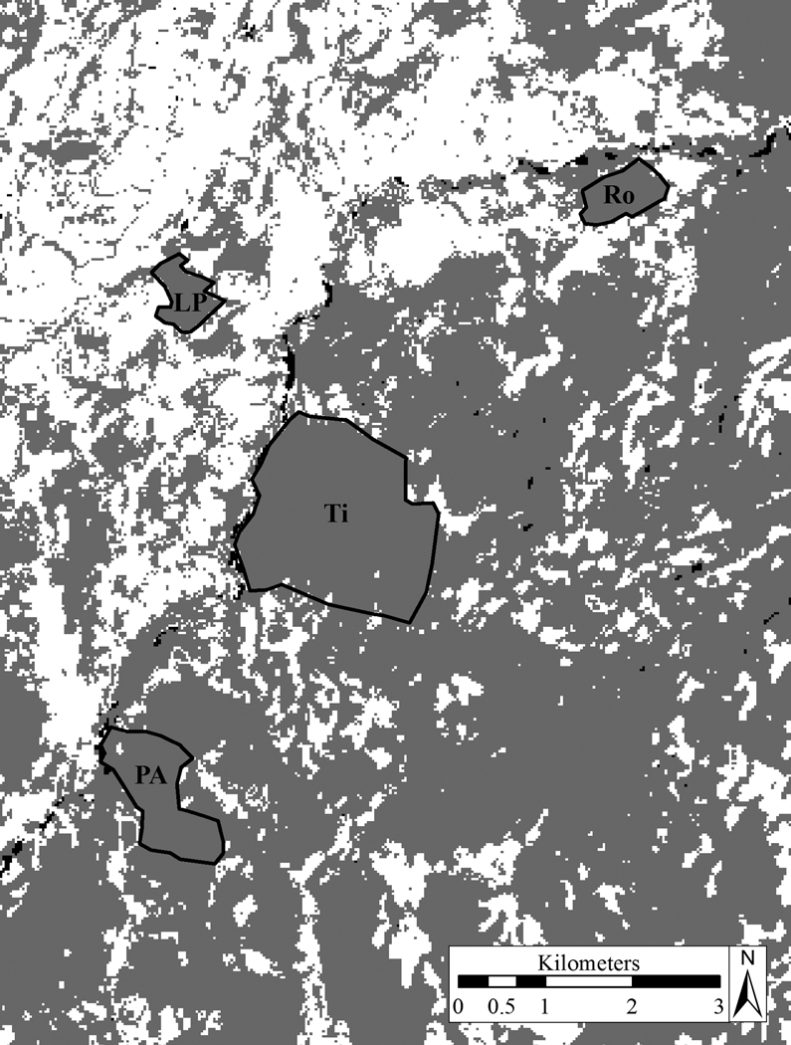
Locations of the four sampled forest fragments in Northern Costa Rica. LP: Las Palmitas, Ro: El Roble, Ti: La Tirimbina, PA: Pozo Azul. *Grey* indicates forested areas, *white* indicates non-forest cover, *black* indicates water (except the fragments’ framing). Modified from [65].

We worked in four forest fragments. Two of these were characterized by small size and high degree of isolation (Las Palmitas, LP; El Roble, Ro). An intermediate (Pozo Azul, PA) and a large fragment (La Tirimbina, Ti) were better connected to neighboring forest patches (Table 1; Fig. 1). The vegetation within the fragments was dominated by primary and old secondary forest. The surrounding matrix was composed of cattle pastures, fruit plantations, urban structures and vegetation that remained after clear-cutting.

**Table 1:** Summary of details on locations and telemetry results of 16 tracked bat individuals.

bat ID	sex	forest patch	fragment size [ha]	HR_tot_	#fixes	com-pactness	#focus areas	observation	
								days	hours
1	M	Ti	412	18.85	199	0.84	2	6	36
2	M	Ro	47	12.46	273	0.79	2	6	36
3	F	Ro	47	10.55	232	0.65	1	6	36
4	m	LP	38	26.91	115	0.50	2	6	31
5	f	LP	38	25.82	182	0.56	2	6	32
6	f	PA	99	5.59	283	0.94	1	7	38
7	m	PA	99	6.96	93	0.74	1	4	23
8	f	Ti	412	3.97	152	0.83	1	3	18
9	m	Ti	412	6.82	211	0.81	1	6	34
10	m	LP	38	5.19	416	0.99	1	6	35
11	m	LP	38	6.19	136	0.85	1	5	30
12	m	Ro	47	5.25	330	0.91	1	6	36
13	f	Ti	412	30.63	121	0.64	2	6	36
14	m	PA	99	6.60	291	0.94	1	6	36
15	f	Ro	47	1.69	258	0.87	1	6	36
16	m	PA	99	6.53	361	0.89	1	6	36

HR_tot_: home range measured as 95% minimum convex polygon; compactness ratio (cf [45]); number of focal foraging areas was identified via LoCoH 50% isopleths

### Study species

We addressed movement patterns of the bat species *Dermanura watsoni* (Phyllostomidae: Stenodermatinae). This small-bodied species feeds on a wide variety of fruits of plants from early to late stages of rainforest succession. More than 50 plant species have been documented to be dispersed by *D*. *watsoni* in Northern Costa Rica [14, 25]. *Dermanura watsoni* is a tent-roosting bat and constructs its roosts by modifying leaves of common rainforest understory plants (e.g., Araceae, Arecaceae, Cyclanthaceae) [28].

### Bat capture and transmitter attachment

We mist-netted bats using eight nets (six nets 12 x 2.5 m, two nets 6 x 2.5 m) set along trails at ground level that were opened at dusk and closed between midnight and 1 am. We complemented mist-netting with capturing animals directly from leaf-tents during the day using a custom made hand net. We kept bats in cotton cloth bags before verification of the species and recording age, sex, and reproductive state. We classified bats as adult when no epiphyseal gaps were visible and the phalangeal-metacarpal joints were knobby [29]. We considered female bats as non-reproductive or in a very early stage of pregnancy when not exceeding a weight of 13 g since 12 to 13 g body weight was a common measure outside the breading season. We selected only males with scrotal testes, which is the case for most adult males during this part of the year [30] or females not exceeding 13 g for telemetry. Pregnant and lactating females were excluded from our study in order to reduce variability in foraging patterns that may be caused by differences in reproductive state.

We used LB-2N radio-transmitters (Holohil Systems) with a position switch that indicated whether the bat was flying or roosting by varying pulse frequency. Total transmitter weight was 0.7 g, corresponding to 5.3–5.8% of the body weight of the 12–13 g bats. This was close to the 5% which were shown to have no impacts on bats [31] and well below the 10% which should not be exceeded if transmitters are carried for more than a few days [32]. We partially trimmed the fur between the shoulder blades and glued the transmitter to the skin (Perma-Type Surgical Cement for 13 bats, Pattex for three bats). The bats were released at the capture site within one hour after capture.

### Radiotelemetry

We tracked a total of 16 individuals (10 males and 6 females) from four forest fragments (Table 1). In four occasions we marked two animals simultaneously (IDs 2&3, 4&5, 6&7, 8&9, respectively). Observation started at the day roost at sunset (around 6 pm) and ended usually at midnight. To ensure that the bat behavior did not differ during the second half of the night, the observation was extended until 6 am during three tracking nights. In order to estimate bat locations via triangulation, two observers followed the bats on foot. Each observer was equipped with an H-Antenna that carried an attached compass and was connected to a Yaesu VR-500 receiver (modified by Wagener Telemetrieanlagen). Furthermore, equipment included a Garmin GPSmap 60cs GPS device, a mobile radio, a digital watch, and a digital voice recorder. We collected bearings to the closest degree in two-minute intervals, coordinated to the second between the two observers. The observation period of individuals varied from three to seven 6-hour-intervals, and in most cases we collected six intervals during a period of five to nine days (Table 1). Transmitters usually fell off after five to ten days. We paused the observation from three nights before to three nights after full moon.

### Data processing

We used the transverse Mercator projection and WGS 1984 datum for working on geographic data. Magnetic declination accounted to only 0.5° and was therefore disregarded. We only considered pairs of bearings with a difference of 15–165° for further analysis. Furthermore, we manually removed all bat positions that were further away from the observers than 400 m as such values were exceeding the transmission range of the transmitters and hence not reliable. We used the software LOAS 4.0 (Ecological Software Solutions LLC) to estimate animal locations. When a bat remained in the same place for more than two minutes, we only kept one data point for further analysis in order to reduce spatial autocorrelation.

### Data analysis

#### Habitat selection

For determination of the total home range (HR_tot_) we calculated 95% minimum convex polygons (MCPs) in Biotas 2.0a (Ecological Software Solutions). For habitat selection analyses we calculated selection ratios *w*_*i*_ in order to rank available habitat categories according to their usage [33]. In absence of selection a value of 1 is expected while values between 0 and 1 indicate negative selection and positive selection is expressed by values larger than 1. We constructed Bonferroni’s confidence intervals (CIs) in order to assess whether *w*_*i*_ selection ratios were significantly different from 1 [33]. We analyzed usage and availability at the individual level (design 3 sensu [34]). Tests were performed in the library adehabitat [35] in R 2.15.1 [36].

We defined “available habitat” individually for each bat as the habitat in a circle around the day roost with a radius corresponding to the maximum distance from the day roost to the line of its 95% MCP (cf [37]). In case several day roosts were used we determined a 100% MCP of the day roost locations and used the center of this polygon as the center for the circle. The “used habitat” corresponds to focal foraging areas (hereafter focus areas) [38]. To define focus areas we used 50% isopleths of fixed k-LoCoH (Local Convex Hulls; [39]). As some bats foraged in highly heterogeneous areas, we chose the LoCoH algorithm for used habitat based on its superior performance in areas with hard boundaries [40]. The square root of the number of locations determined the parameter k, which sets the number of neighbors that are included in the construction of the convex hulls for each position [40].

On-site inspection and aerial photographs (provided by La Tirimbina Rainforest Center) allowed for the categorization of available and used habitat in ArcGIS 10. During on-site inspections we screened a selection of extra forest sites visited by bats for possible food plants. We combined the collected land-cover information with imagery from Google Earth that was updated for the region in March 2011. For the calculation of selection ratios we attributed land-cover types to the following categories based on structural diversity. “Natural” forest was characterized by forest fragments of late secondary or primary forest with mainly closed canopy and understory present. “Degraded” forest denoted early successional forest stages or loose tree aggregations without closed canopy or understory and linear structures, e.g., remnant gallery forests. “Farmland” indicated fruit plantations (mainly pineapple) and abandoned agricultural land. Cattle “pastures” were characterized by grassland with scattered single trees. “Urban” areas combined non-natural anthropogenic structures such as roads and building.

#### Variability of daily ranges

Daily ranges of bats could vary substantially among individuals and also between nights for the same individual. To examine this variability, we fitted mixed models using the library nlme [41] implemented in the software R 2.15.1. We defined the daily range (95% MCP of the area covered during one 6 h observation session) as the response variable. For this purpose we selected three nights per bat with the highest contact times which exceeded 85% of the observation time. For two wider ranging bats (IDs 3 & 4) we included nights below that threshold in which ranging patterns matched behavior of foregoing nights, but commuting flights over impassable terrain did not allow for reaching the 85%. For four bats we had adequate information for only two nights.

Prior to fitting the model we applied a logarithmic transformation on the response variable daily range in order to achieve normal distribution and data homogeneity. The fixed variables included the proportion of degraded forest (disturbance) within the daily range to test for a relationship between range size and land-cover quality. The percentage of the illuminated surface of the moon entered in order to test for a possible effect of lunar phobia. We added the sex of the tracked bat since males and females may behave differently [42]. The sampling day entered as a numeric value that increases in the course of the sampling interval in order to test for changes in ranging behavior over time. We centered the fixed variables and checked for multicollinearity using variance inflation factors (VIFs) in the R library AED [43, 44]. Later versions of R may alternatively use the package car for calculating VIFs. Finally, we added the bat identity as a random intercept to the model in order to account for repeated measurements for individual bats.

For model selection we chose the ‘all-subset approach’ as described in [45] based on the corrected Akaike’s information criterion (AIC_c_) in the R library MuMIn [46]. We performed model averaging, which is well suited to obtain parameter estimates in all-subset model approaches [45], based on a top-model set of Δ2 AIC_c_ as cut-off [47]. Conditional R²-values (R²_c_) [48] were measured to account for the variation explained by both fixed and random effects for all models in the averaged set in the R library MuMIn [46].

#### Home range geometry

We established a measure of the compactness of the total home range for every individual bat (cf [49]). This compactness ratio gives an impression of the home range shape. We derived the compactness ratio by calculating the perimeter of an individual bat’s 95% MCP and dividing the area of this polygon by the area of a hypothetical circle that has the same perimeter (MCP geometry was retrieved from ArcGIS 10). A nearly circular MPC will result in a ratio close to 1, whereas lower values describe home range shapes that are increasingly linear. In order to investigate whether home range shape and size influence each other, we calculated a Pearson correlation between HR_tot_ and the corresponding compactness ratios. We log-transformed HR_tot_ in order to achieve normal distribution (verified by Shapiro-Wilk-tests; α–level = 0.2).

In five cases the 50% LoCoH isopleths were not restricted to a particular site, but revealed two spatially separate focus areas for an individual bat. In these instances we assigned the radio-fixes within the focus areas to the beforehand mentioned land-cover categories (natural, degraded, farmland, pasture, and urban). Then we used Fisher’s Exact tests to test for each bat (n = 5), whether there were differences in the distribution of fixes over the land-cover categories between the “near” focus area, which was close to the day roost, and the “far” focus area, which was several hundred meters apart. Subsequently, a sequential Bonferroni correction accounted for multiple testing.

### Permits and animal welfare

The forest fragments we worked in were privately owned and access was granted by the landowners. All work on live animals followed the guidelines of the Society of Mammologists (ASM) for research on wild mammals [50] and was approved by the “Ministerio de Ambiente, Energía y Telecomunicaciones de Costa Rica” (MINAET). Transmitter attachment for radio-telemetry conformed to common rules regarding transmitter weight in relation to body mass [33]. All permits were obtained from J. Guevara at MINAET (Resolutions: 004–2011-SINAC, 128–2011-SINAC).

## Results

### Roosting behavior

All day roosts of *D*. *watsoni* were situated within natural forests. The bats exclusively roosted in modified leaf tents that belonged to the plant families Arecaceae, Cyclanthaceae, Rubiaceae, or Araceae. Bats always roosted within the same forest fragment during the observation period and returned either to the same leaf tent or roosted in close proximity (less than 50 m).

### Variability in ranging patterns

After the initial inspection of the data from all radio-fixes collected during 76 tracking nights, we found 3653 to be reliable bat encounters during roosting and foraging. Total home range (95% MCP) averaged at 11.3 ± 9.1 ha and varied among individual bats from 1.7 to 30.6 ha (Table 1). We also observed considerable variability among daily ranges for single individuals.

The fixed variables that entered the mixed models had variance inflation factors that were below 3 indicating there was no collinearity among those variables [43] (S1 Table). The all-subset model approach generated a total of 16 models (S2 Table). The Δ2 AICc model set contained only two models and indicated that forest disturbance was the most important variable to explain variability in daily ranges since it was the single parameter in the top model and also present in the second model of the averaged set (Table 2). Daily range size increased with increasing amounts of degraded forest (Table 3). Daily ranges tended to decrease in the course of the sampling period. However, the parameter sampling day was weighted much lower than the proportion of degraded forest and the conditional standard error was high (Table 3). Sex and moonshine seemed to have no effect, since they were not included in the top model set. Furthermore, those parameters were weighted fairly low and standard errors were high when averaging over all subset models (S3 Table).

**Table 2:** Multi-model inference based on the Δ2 AIC_**c**_ candidate model set for the effects of the proportion of degraded forest (Disturbance) and sampling day (Day) on daily range size in ***Dermanura watsoni*** (n = 44).

Candidate model	*K*	*R^2^*_*c*_	AIC_c_	ΔAIC_c_	*w*	*w*_acc_
Disturbance	4	0.65	69.2	0	0.66	0.66
Disturbance + Day	5	0.66	70.5	1.32	0.34	1.00

*R^2^*_*c*_ (conditional *R^2^*) is the variance explained by both fixed and random effects [44]. The identity of 16 bat individuals was fitted as random intercept.

**Table 3:** Summary of the parameters proportion of degraded forest (Disturbance) and sampling day (Day) that were included in the Δ2 AIC_**c**_ model set, averaged over all subset models.

Fixed variable	Estimate	Unconditional SE	Rel. importance	Confidence intervals	
				Lower	Upper
(Intercept)	10.57	0.10		10.35	10.78
Disturbance	3.22	0.89	0.95	1.39	5.04
Day	-0.43	0.39	0.38	-1.24	0.38

We tested whether size variability in total home range was related to geometric changes in shape. A Pearson correlation between log HR_tot_ and the compactness ratios showed a highly significant negative association among the two parameters, indicating that larger home ranges become increasingly linearly shaped (r = -0.73; 95% CI = -0.90, -0.37; *p* = 0.001).

### Habitat selection

The analysis for habitat selection revealed that the bats did not move randomly but foraged selectively within the available habitat (χ^2^ = 340.8, df = 12, p < 0.001). Natural forest was positively selected (*w*_*i*_ > 1, CI = 1.030, 1.471) while pastures were negatively selected (*w*_*i*_ < 1, CI = 0.002, 0.407; Table 4). The *w*_*i*_ selection ratios for degraded forest and farmland were smaller than 1, but the confidence intervals contained the value 1 indicating that *w*_*i*_ was not significantly different from 1 [33].

**Table 4:** Habitat selection by 16 radio-tracked bat individuals based on selection ratios (***w***_**i**_) for five different land-cover types.

			Bonferroni’s confidence intervals	
	*w*_*i*_	SE	Lower	Upper
Natural	1.25	0.08	1.030	1.471
Degraded	0.86	0.32	0.000^a^	1.791
Farmland	0.54	0.21	0.000^a^	1.140
Pasture	0.2	0.07	0.002	0.407
Urban	0	0	-	-

Standard errors (SE) and Bonferroni’s confidence intervals are shown.

^a^ negative confidence limits have been replaced by 0.000 [33]

**Fig 2:**
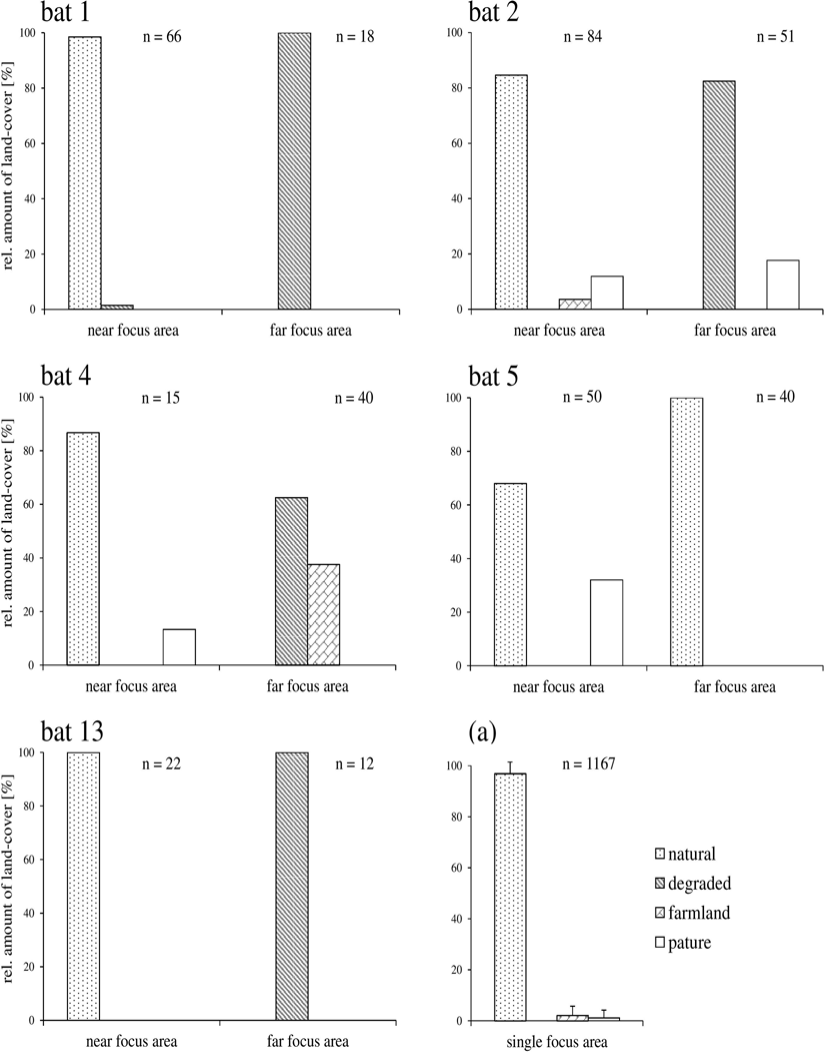
Land-cover composition of focal foraging areas of 16 bat individuals. Bat IDs refer to table 1. Near focus areas were located closer to the day roost. Far focus areas were visited by longer distance commutes. (a) averaged values of 11 bat individuals which foraged on a single focus area. n indicates the number of radio fixes.

In eleven bats total home ranges comprised a focus area that contained the day roost(s) of the respective bat or was located close by. However, in five individuals the 50% LoCoH isopleths showed a second, spatially separated focus area, on average 660 m (range: 330 to 1050 m) away from the particular day roost. Commutes between focus areas extended about 200 to 340 m beyond the forest border into the matrix. Those bats used to forage at first within the near focus area close to the day roost and performed one to two foraging bouts to the far focus area within a six-hour interval. The Fisher’s Exact tests revealed highly significant differences between the habitat categories that corresponded to bat locations within the near vs. the far focus area, respectively, for all five bats after sequential Bonferroni correction (*p* < 0.001 in all five cases; lowest adjusted α–level: 0.05/5 = 0.01). In all five individuals the focus area that was situated closer to the day roost was strongly dominated by natural forest, as it was in the bats with only one focus area, whereas the second patch was dominated by natural forest only in a single individual, and by degraded forest in four individual bats (Fig. 2). Inspection of such degraded sites where bats were foraging revealed that pioneer plants such as *Cecropia* sp. and *Solanum* sp. were present.

## Discussion

Our study provides insight into how an abundant frugivorous bat species deals with a highly heterogeneous, anthropogenically modified landscape. Roosting and foraging behavior showed that *D*. *watsoni* strongly relies on habitat remnants that are dominated by natural vegetation. However, the bats are also capable of entering the matrix and performing foraging bouts to degraded areas by traversing the matrix. This highlights their potential to functionally connect elements in a fragmented landscape by commuting between natural and degraded sites.

### Roosting requirements

We observed that individuals of *D*. *watsoni* spent the day roosting inside a natural forest fragment, even when foraging at night outside the fragment in degraded areas. A similar dependence for forest habitats for roosting in fragmented areas has been documented for the phyllostomid bats *Artibeus lituratus* and *Carollia perspicillata* [23]. By contrast, *Uroderma bilobatum* frequently roosts in leaf-tents within anthropogenic areas such as gardens or plantations as it even modifies leafs of cultivated coconut palms to tents [51]. Although *D*. *watsoni* is able to construct its roosts from modifying leafs of a wide variety of plant species [28], we observed it roosting in leafs of forest understory plants (Arecaceae, Cyclanthaceae, Rubiaceae) or Epiphytes (Araceae) that were widely missing within the degraded areas outside the forest fragments. This preference for certain plants that are only present within natural forest shows that *D*. *watsoni* strongly relies on fairly natural habitats for roost construction and highlights the value of natural forest remnants for bats in agricultural landscapes.

### Foraging behavior

Selection ratios revealed that the bats foraged selectively and positively selected natural forest. A similar pattern was reported for the phyllostomid bats *A*. *lituratus* and *C*. *perspicillata* that preferred restored forest areas and avoided early successional forest and anthropogenically modified land [23]. In our study, farmland and pastures that contained only few scattered trees do not seem to be very attractive to *D*. *watsoni*, as they offer only few resources. Even if single trees in pastures (e.g., fig trees) might bear ripe fruits, predation risk while exploiting those resources might be elevated, as potential predators for bats, such as birds of prey, are frequently associated with forest edges or matrix [52, 53]. This risk might be further increased by the lack of suitable roosting sites for safely consuming fruits in open areas [14].

Foraging bouts to degraded areas could only be observed in a subset of individuals. In general, forest disturbance had the highest weight in our models and larger daily ranges were associated with increasing relative amounts of degraded forest. Movement studies on bats and primates already demonstrated a negative relationship between habitat quality and home range size, leading to smaller home ranges in mature forest [54, 55]. Similarly, two phyllostomid bat species responded to high resource availability with a decrease in home range size [56, 57]. However, all individuals observed in our study were roosting and hence started their nightly foraging in natural forest fragments. Two of these (La Tirimbina and El Roble) and the nearby biological station La Selva were surveyed during dietary studies on *D*. *watsoni*, revealing more than 50 species of small and larger seeded plants used as food resources [14, 58]. This broad food spectrum indicates that food resources should usually not be scarce for *D*. *watsoni* in the forest types we worked in. Still, instead of foraging on small areas within natural forest our data suggest directed commuting flights from natural forest towards degraded areas in some individuals thus enlarging the daily range. Directional commuting behavior is indicated by the geometry of the home ranges that become more linearly shaped with increasing size, as shown by the compactness ratio that was negatively correlated with home range size. The destination of such commuting flights often was a second focus area that differed in habitat composition from the first one around the day roost and was mostly dominated by degraded forest. This highlights two different foraging behaviors: Either foraging on a rather small area inside a forest fragment or performing, in addition to feeding around the day roost, commutes to remote degraded sites that are several hundred meters to more than one kilometer away. The motivation of an animal to move into the matrix strongly depends on the emerging risks and benefits [8], where species entering more open areas may perceive elevated predation risk [59, 60]. However, elevated resource availability in combination with lower levels of competition may offset this risk of such long-distance commutes and the exploitation of extra-forest food resources might in fact increase the carrying capacity for *D*. *watsoni*, which is one of the most abundant species in the region [26].

### Potential benefits for mosaic landscapes

While commuting between two focus areas, bats extended foraging to areas within the matrix which were located up to 340 m beyond the forest border. Such movements highlight the bats’ potential for conservation and restoration processes. Tree populations in fragmented areas face the risk of losing genetic diversity as a result of missing seed dispersal among fragments [61], as human encroachment (e.g., land-use changes and hunting) affects especially the larger mammalian dispersers such as agoutis or peccaries [6, 62]. Our observations on foraging movements show that even small bats may contribute to long-distance seed dispersal in degraded areas. Furthermore, commutes between natural and degraded areas confirm assumptions that bats may not only foster establishment of early succession in pastures, but also support the reestablishment of primary forest species in more degraded areas [63].

## Conclusion

Our results show that the small frugivorous bat *D*. *watsoni* can not only persist in anthropogenically dominated landscape mosaics, but may also functionally connect natural and degraded habitat. Foraging bouts to degraded forests facilitate long-distance seed dispersal from natural to degraded sites, and hence foster regeneration processes. However, the presence of natural forest remnants is crucial for the persistence, as roosting requires natural habitat patches. Those findings underpin the hypothesis that forest fragments are of high value for phyllostomid bats [64] with behavioral data and in turn emphasize the important ecosystem functions of phyllostomid bats within mosaic landscapes. Still, to preserve those valuable organisms, attractive fruit plants must not be scarce and remote from natural forest patches, and habitat connectivity needs to be maintained as pointed out by the relatively short flight distances through matrix and the avoidance of open areas recorded in our telemetry observations. Conservation measures that aim at preserving natural forest remnants and reconnect them are urgently needed, as habitat connectivity is also associated with genetic diversity in *D*. *watsoni* populations and negative effects may manifest within few decades [26].

## Supporting Information

S1 Table Assessment of multicollinearity of the fixed variables of the full model by variance inflation factors. Values below 3 indicate no collinearity among variables [39]. Fixed variables are the proportion of degraded forest (Disturbance), the sampling day (Day), the proportion of the illuminated moon surface (Moon), and the sex of the bat individual (Sex).
(DOCX)

S2 Table Model set generated by the ‘all-subset approach’ using mixed models. We assessed the effect of the proportion of degraded forest within the daily range (Disturbance), sex of the bat individual (Sex), proportion of the illuminated surface of the moon (Moon), and the day of the sampling period (Day) on the size of daily ranges. The identity of 16 bat individuals was fitted as a random intercept.
(DOCX)

S3 Table Summary of the effects of parameters on daily range size, averaged over all subset models. Fixed variables include the proportion of degraded forest (Disturbance), the sampling day (Day), the proportion of the illuminated moon surface (Moon), and the sex of the bat individual (Sex). The identity of 16 bat individuals was fitted as a random intercept.
(DOCX)

S4 Table Primary data for fitting mixed models on variability of daily ranges. (DOCX)

S5 Table Data on available and used habitat that entered the habitat selection analysis. (DOCX)
