# Supplementary material for: Frugivorous Bats Maintain Functional Habitat Connectivity in Agricultural Landscapes but Rely Strongly on Natural Forest Fragments
Source: PLoS One. 2015 Apr 1;10(4):e0120535. doi: 10.1371/journal.pone.0120535 (PMC4382216; doi:10.1371/journal.pone.0120535)
Supplement: S1 Table — Values below 3 indicate no collinearity among variables [39]. Fixed variables are the proportion of degraded forest (Disturbance), the sampling day (Day), the proportion of the illuminated moon surface (Moon), and the sex of the bat individual (Sex). (DOCX) [file pone.0120535.s001.docx]

**Table S1.**

| Fixed variable | Variance inflation factors |
| --- | --- |
| Disturbance | 2.255 |
| Day | 1.684 |
| Moon | 1.539 |
| Sex | 1.006 |
