# Supplementary material for: Frugivorous Bats Maintain Functional Habitat Connectivity in Agricultural Landscapes but Rely Strongly on Natural Forest Fragments
Source: PLoS One. 2015 Apr 1;10(4):e0120535. doi: 10.1371/journal.pone.0120535 (PMC4382216; doi:10.1371/journal.pone.0120535)
Supplement: S2 Table — We assessed the effect of the proportion of degraded forest within the daily range (Disturbance), sex of the bat individual (Sex), proportion of the illuminated surface of the moon (Moon), and the day of the sampling period (Day) on the size of daily ranges. The identity of 16 bat individuals was fitted as a random intercept. (DOCX) [file pone.0120535.s002.docx]

**Table S2.**

| Candidate model*^a^* | *k* | AIC_c_ | ΔAIC_c_ | *w* | *w*_acc_ |
| --- | --- | --- | --- | --- | --- |
| Disturbance | 4 | 69.17 | 0 | 0.36 | 0.36 |
| Disturbance + Day | 5 | 70.49 | 1.32 | 0.19 | 0.55 |
| Disturbance + Moon | 5 | 71.38 | 2.21 | 0.12 | 0.67 |
| Disturbance + Sex | 5 | 71.7 | 2.54 | 0.10 | 0.78 |
| Disturbance + Day +Moon | 6 | 72.28 | 3.11 | 0.08 | 0.85 |
| Disturbance + Day + Sex | 6 | 73.16 | 3.99 | 0.05 | 0.90 |
| Disturbance + Sex +Moon | 6 | 74.04 | 4.88 | 0.03 | 0.94 |
| Day + Moon | 5 | 74.48 | 5.32 | 0.03 | 0.96 |
| Disturbance + Day + Sex + Moon^a^ | 7 | 75.08 | 5.91 | 0.02 | 0.98 |
| Day | 4 | 76.79 | 7.62 | 0.01 | 0.99 |
| Day + Sex + Moon | 6 | 77.16 | 7.99 | 0.01 | 0.99 |
| Moon | 4 | 79.32 | 10.15 | < 0.01 | 1 |
| Day + Sex | 5 | 79.33 | 10.17 | < 0.01 | 1 |
| (Intercept) | 3 | 80.36 | 11.19 | < 0.01 | 1 |
| Sex + Moon | 5 | 81.87 | 12.7 | < 0.01 | 1 |
| Sex | 4 | 82.74 | 13.57 | < 0.01 | 1 |

*^a^* Full model
