# Supplementary material for: Frugivorous Bats Maintain Functional Habitat Connectivity in Agricultural Landscapes but Rely Strongly on Natural Forest Fragments
Source: PLoS One. 2015 Apr 1;10(4):e0120535. doi: 10.1371/journal.pone.0120535 (PMC4382216; doi:10.1371/journal.pone.0120535)
Supplement: S3 Table — Fixed variables include the proportion of degraded forest (Disturbance), the sampling day (Day), the proportion of the illuminated moon surface (Moon), and the sex of the bat individual (Sex). The identity of 16 bat individuals was fitted as a random intercept. (DOCX) [file pone.0120535.s003.docx]

**Table S3.**

| Fixed variable | Estimate | Unconditional SE | Rel. importance | Confidence intervals | |
| --- | --- | --- | --- | --- | --- |
|  |  |  |  | Lower | Upper |
| (Intercept) | 10.58 | 0.17 |  | 10.22 | 10.94 |
| Disturbance | 3.13 | 0.95 | 0.95 | 1.18 | 5.07 |
| Day | -0.53 | 0.44 | 0.38 | -1.42 | 0.37 |
| Moon | -0.32 | 0.41 | 0.28 | -1.15 | 0.51 |
| Sex | -0.031 | 0.22 | 0.21 | -0.50 | 0.44 |
