# Supplementary material for: Frugivorous Bats Maintain Functional Habitat Connectivity in Agricultural Landscapes but Rely Strongly on Natural Forest Fragments
Source: PLoS One. 2015 Apr 1;10(4):e0120535. doi: 10.1371/journal.pone.0120535 (PMC4382216; doi:10.1371/journal.pone.0120535)
Supplement: S4 Table — (DOCX) [file pone.0120535.s004.docx]

**Table S4.**

| bat  ID | sex | date | sampling  day | site | daily range  [m²] | degraded  (proportion) | moon  (proportion) |
| --- | --- | --- | --- | --- | --- | --- | --- |
| 1 | m | 03II | 1 | Ti | 126455 | 0.442 | 0 |
| 1 | m | 04II | 2 | Ti | 88320 | 0.415 | 6.7 |
| 1 | m | 05II | 3 | Ti | 132192 | 0.485 | 13.3 |
| 2 | w | 09II | 7 | Ro | 81214 | 0.179 | 40 |
| 2 | w | 10II | 8 | Ro | 49071 | 0.259 | 46.7 |
| 2 | w | 11II | 9 | Ro | 55124 | 0.178 | 53.3 |
| 3 | m | 08II | 6 | Ro | 88357 | 0.076 | 33.3 |
| 3 | m | 10II | 8 | Ro | 67848 | 0.115 | 46.7 |
| 3 | m | 11II | 9 | Ro | 24531 | 0 | 53.3 |
| 4 | m | 26II | 24 | LP | 16128 | 0.096 | 42.9 |
| 4 | m | 27II | 25 | LP | 104329 | 0.118 | 35.7 |
| 5 | w | 26II | 24 | LP | 126439 | 0.134 | 42.9 |
| 5 | w | 28II | 26 | LP | 137127 | 0.049 | 28.6 |
| 6 | w | 11III | 37 | PA | 40733 | 0 | 46.7 |
| 6 | w | 12III | 38 | PA | 35165 | 0 | 53.3 |
| 6 | w | 13III | 39 | PA | 32493 | 0 | 60 |
| 7 | m | 14III | 40 | PA | 19713 | 0 | 66.7 |
| 7 | m | 15III | 41 | PA | 30041 | 0 | 73.3 |
| 8 | w | 23III | 49 | Ti | 21621 | 0 | 73.3 |
| 8 | w | 24III | 50 | Ti | 19507 | 0 | 66.7 |
| 8 | w | 27III | 53 | Ti | 30469 | 0.145 | 46.7 |
| 9 | m | 25III | 51 | Ti | 49436 | 0 | 60 |
| 9 | m | 30III | 56 | Ti | 29525 | 0 | 26.7 |
| 9 | m | 31III | 57 | Ti | 48011 | 0 | 20 |
| 10 | m | 04IV | 61 | LP | 23895 | 0 | 6.7 |
| 10 | m | 07IV | 64 | LP | 28306 | 0 | 26.7 |
| 10 | m | 09IV | 66 | LP | 19751 | 0 | 40 |
| 11 | m | 12IV | 69 | LP | 36869 | 0 | 60 |
| 11 | m | 13IV | 70 | LP | 23250 | 0 | 66.7 |
| 11 | m | 14IV | 71 | LP | 11029 | 0 | 73.3 |
| 12 | m | 25IV | 82 | Ro | 33982 | 0 | 53.3 |
| 12 | m | 26IV | 83 | Ro | 43653 | 0 | 46.7 |
| 12 | m | 27IV | 84 | Ro | 26894 | 0 | 40 |
| 13 | w | 01V | 88 | Ti | 69442 | 0.232 | 13.3 |
| 13 | w | 03V | 90 | Ti | 46853 | 0.098 | 0 |
| 14 | m | 11V | 98 | PA | 30995 | 0 | 57.1 |
| 14 | m | 12V | 99 | PA | 41644 | 0 | 64.3 |
| 14 | m | 13V | 100 | PA | 47453 | 0 | 71.4 |
| 15 | w | 27V | 114 | Ro | 11686 | 0 | 33.3 |
| 15 | w | 28V | 115 | Ro | 15593 | 0 | 26.7 |
| 15 | w | 29V | 116 | Ro | 13322 | 0 | 20 |
| 16 | m | 05VI | 123 | PA | 28799 | 0 | 28.6 |
| 16 | m | 06VI | 124 | PA | 33255 | 0 | 35.7 |
| 16 | m | 07VI | 125 | PA | 65050 | 0 | 42.9 |
