# Supplementary material for: Frugivorous Bats Maintain Functional Habitat Connectivity in Agricultural Landscapes but Rely Strongly on Natural Forest Fragments
Source: PLoS One. 2015 Apr 1;10(4):e0120535. doi: 10.1371/journal.pone.0120535 (PMC4382216; doi:10.1371/journal.pone.0120535)
Supplement: S5 Table — (DOCX) [file pone.0120535.s005.docx]

**Table S5.**

| bat ID | land cover (available / used) | | | | |
| --- | --- | --- | --- | --- | --- |
|  | natural | degraded | pasture | farmland | urban |
| 1 | 387024 / 65 | 298577 / 19 | 9158 / 0 | 0 / 0 | 0 / 0 |
| 2 | 292976 / 71 | 124298 / 42 | 313241 / 19 | 23000 / 3 | 10075 / 0 |
| 3 | 238198 / 103 | 112102 / 0 | 279936 / 12 | 23000 / 0 | 21420 / 0 |
| 4 | 716633 / 13 | 666985 / 25 | 898721 / 2 | 808474 / 15 | 430393 / 0 |
| 5 | 695700 / 74 | 201818 / 0 | 565309 / 16 | 472830 / 0 | 351701 / 0 |
| 6 | 135067 / 102 | 2301 / 0 | 0 / 0 | 1250 / 0 | 0 / 0 |
| 7 | 164738 / 19 | 5422 / 0 | 38421 / 0 | 0 / 0 | 0 / 0 |
| 8 | 936832 / 75 | 40318 / 0 | 1417 / 0 | 89531 / 0 | 0 / 0 |
| 9 | 183822 / 71 | 0 / 0 | 0 / 0 | 0 / 0 | 0 / 0 |
| 10 | 60844 / 170 | 1937 / 0 | 5996 / 0 | 12935 / 24 | 0 / 0 |
| 11 | 108101 / 66 | 0 / 0 | 18335 / 0 | 0 / 0 | 0 / 0 |
| 12 | 81582 / 161 | 0 / 0 | 2879 / 0 | 0 / 0 | 0 / 0 |
| 13 | 711285 / 22 | 278455 / 12 | 23006 / 0 | 0 / 0 | 10240 / 0 |
| 14 | 135223 / 94 | 0 / 0 | 27657 / 1 | 0 / 0 | 0 / 0 |
| 15 | 55909 / 127 | 0 / 0 | 17403 / 0 | 0 / 0 | 0 / 0 |
| 16 | 197315 / 137 | 0 / 0 | 42605 / 0 | 0 / 0 | 3606 / 0 |
